# Impact of Age on the Efficacy and Safety of Alirocumab in Patients with Heterozygous Familial Hypercholesterolemia

**DOI:** 10.1007/s10557-019-06852-6

**Published:** 2019-02-08

**Authors:** Henry N. Ginsberg, Jaakko Tuomilehto, G. Kees Hovingh, Bertrand Cariou, Raul D. Santos, Alan S. Brown, Santosh K. Sanganalmath, Andrew Koren, Desmond Thompson, Frederick J. Raal

**Affiliations:** 1 Irving Institute for Clinical and Translational Research Columbia University, Columbia University Vagelos College of Physicians and Surgeons, Columbia University, 622 West 168 Street, PH-10, New York, NY 10032 USA; 2 Public Health Solutions, National Institute for Health and Welfare, Helsinki, Finland; 3 Diabetes Research Group, King Abdulaziz University, Jeddah, Saudi Arabia; 4 Department of Vascular Medicine, Academic Medical Center, University of Amsterdam, Amsterdam, The Netherlands; 5 l’institut du thorax, Department of Endocrinology, CHU Nantes, CIC1413 INSERM, Nantes, France; 6 Lipid Clinic Heart Institute (InCor), University of São Paulo Medical School Hospital, São Paulo, Brazil; 7 Hospital Israelita Albert Einstein, São Paulo, Brazil; 8 Division of Cardiology, Advocate Lutheran General Hospital, Park Ridge, IL USA; 9 Regeneron Pharmaceuticals, Inc., Tarrytown, NY USA; 10 Sanofi, Bridgewater, NJ USA; 11 Faculty of Health Sciences, University of the Witwatersrand, Johannesburg, South Africa

**Keywords:** Cardiovascular disease prevention, Cholesterol-lowering drugs, Clinical trials, LDL-C, PCSK9

## Abstract

**Purpose:**

This post-hoc analysis examined whether age modified the efficacy and safety of alirocumab, a PCSK9 inhibitor, in patients with heterozygous familial hypercholesterolemia (HeFH), using pooled data from four 78-week placebo-controlled phase 3 trials (ODYSSEY FH I, FH II, LONG TERM, and HIGH FH).

**Methods:**

Data from 1257 patients with HeFH on maximally tolerated statin ± other lipid-lowering therapies were analyzed by an alirocumab dose regimen and by age subgroups (18 to < 45, 45 to < 55, 55 to < 65, and ≥ 65 years). In the FH I and II trials, patients received 75 mg subcutaneously every 2 weeks (Q2W), with dose increase to 150 mg Q2W at week 12 if week 8 low-density lipoprotein cholesterol (LDL-C) was ≥ 70 mg/dl. In HIGH FH and LONG TERM, patients received 150 mg alirocumab Q2W.

**Results:**

Baseline characteristics were similar between treatment groups across all age groups; the proportion of males decreased whereas the proportion of patients with coronary heart disease, diabetes, hypertension, and declining renal function increased with increasing age. Mean LDL-C reductions at week 24 were consistent across age groups (50.6–61.0% and 51.1–65.8% vs. placebo for the 75/150 and 150 mg alirocumab dose regimens, respectively; both non-significant interaction *P*-values). Treatment-emergent adverse events occurred in similar frequency in alirocumab- and placebo-treated patients regardless of age, except for injection-site reactions, which were more common in alirocumab than placebo but declined in frequency with age.

**Conclusions:**

Alirocumab treatment resulted in significant LDL-C reductions at weeks 12 and 24 and was generally well tolerated in patients with HeFH across all age groups studied.

**Electronic supplementary material:**

The online version of this article (10.1007/s10557-019-06852-6) contains supplementary material, which is available to authorized users.

## Introduction

Patients with heterozygous familial hypercholesterolemia (HeFH) are characterized by high levels of low-density lipoprotein cholesterol (LDL-C) and elevated risk of atherosclerotic cardiovascular disease (ASCVD), most commonly resulting from autosomal dominant mutations in genes of the LDL receptor pathway (e.g., the *LDLR*, *APOB*, and *PCSK9* genes) [1, 2]. Early diagnosis and treatment are crucial to reduce the risk of cardiovascular (CV) events; however, as children and adolescents are asymptomatic (elevated LDL-C may be the only clinical characteristic), diagnosis at a young age may only occur if there is a strong family history or if the condition is severe and clinical signs such as tendon xanthoma are evident [1]. Advancing age and/or comorbidities (for example, hypertension, type 2 diabetes, and renal dysfunction) further increase the risk for cardiovascular disease (CVD) and CV events [3, 4]. For patients with HeFH, LDL-C goals of < 70 or < 100 mg/dl have been recommended by the European Society of Cardiology (ESC)/European Atherosclerosis Society (EAS), the National Lipid Association, and most recently, the updated guidelines from the American Heart Association and American College of Cardiology, for those who are at very high or high CV risk, respectively [3–5].

Statin therapy is generally recommended as first-line treatment to reduce LDL-C levels [3–5]. However, individuals with HeFH often require additional LDL-C-lowering beyond that achieved with high-intensity statins, including addition of ezetimibe, and/or bile acid sequestrants, to achieve LDL-C goals. Proprotein convertase subtilisin/kexin type 9 (PCSK9) inhibitors may be considered for individuals who require additional LDL-C reduction [3–6].

The PCSK9 inhibitor alirocumab is a human monoclonal antibody that blocks the extra-cellular activity of PCSK9. Treatment with alirocumab results in significant LDL-C reductions in adult patients with clinical ASCVD and HeFH treated with maximally tolerated doses of statins ± other lipid-lowering therapies [7–9]. It is unknown, however, whether age modifies the LDL-C-lowering efficacy and safety of alirocumab in adult patients with HeFH. Therefore, using pooled data from four ODYSSEY phase 3 trials, this post-hoc analysis investigated the impact of age on the efficacy and safety of alirocumab in patients with HeFH.

## Methods

Data from four double-blind, randomized, placebo-controlled, 78-week ODYSSEY phase 3 studies were pooled: FH I (NCT01623115) [7], FH II (NCT01709500) [7], LONG TERM (NCT01507831) [9], and HIGH FH (NCT01617655) [8]. The methods and results of each trial have been published previously [7–9]. The trials included patients with HeFH who were on maximally tolerated statin ± other lipid-lowering therapies. Patients with HeFH and LDL-C ≥ 70 mg/dl (in those with a history of CVD) or ≥ 100 mg/dl (without a history of CVD) at screening were enrolled in the FH I and FH II studies. Patients with HeFH and LDL-C levels ≥ 160 mg/dl at screening were included in the HIGH FH trial. The LONG TERM trial included patients with HeFH or hypercholesterolemia and established coronary heart disease (CHD), or patients with LDL-C ≥ 70 mg/dl and a CHD risk equivalent at screening. Only patients with HeFH from the LONG TERM trial were included in this analysis. In FH I and FH II, patients were randomized 2:1 to alirocumab 75 mg every 2 weeks (Q2W) (with possible alirocumab dose increase to 150 mg Q2W at week 12 if LDL-C ≥ 70 mg/dl [1.8 mmol/l] at week 8), or placebo. In LONG TERM and HIGH FH, patients were randomized 2:1 to receive alirocumab 150 mg Q2W or placebo. Alirocumab 75 mg, 150 mg, and placebo were administered subcutaneously using a 1-mL volume injection.

In this analysis, efficacy and safety were assessed in subgroups stratified by age (18 to < 45, ≥ 45 to < 55, ≥ 55 to < 65, and ≥ 65 years). Intention-to-treat analysis (ITT) was used in the evaluation of efficacy endpoints [7–9]. Data were pooled by alirocumab dose regimen trials (75/150 mg Q2W vs. placebo in the FH I and FH II trials, and 150 mg Q2W vs. placebo in the LONG TERM and HIGH FH trials).

Efficacy endpoints included the percentage change in LDL-C from baseline to week 12 and week 24 for each pool stratified by age. LDL-C was calculated using the Friedewald formula in these trials, except when triglycerides (TGs) > 400 mg/dl when LDL-C was determined by beta quantification; however, values determined by beta quantification were excluded in the present analysis. For each pool, additional efficacy endpoints included percentage change in LDL-C from baseline to week 24 stratified by age and HeFH genetic status, as well as reductions in other lipids and lipoproteins from baseline to week 24 stratified by age. In the four phase 3 trials, diagnosis of HeFH was confirmed by either genotyping (44.6% of patients in the ITT population), or clinical criteria (55.4%), as indicated in the patients’ medical records. However, information on mutation types or methods (e.g., whole gene sequencing or number of single nucleotide polymorphisms used) were not detailed in the case report forms. For patients who had not been genotyped, clinical diagnosis was based on either World Health Organization/Dutch Lipid Clinical Network criteria with a score > 8 points, or meeting Simon Broome criteria for definite FH [7].

Safety was analyzed assessing rates of treatment-emergent adverse events (TEAEs), serious adverse events, TEAEs leading to death or treatment discontinuation, and TEAEs reported in at least 5% of patients by age group.

## Statistical Analyses

Efficacy endpoints were evaluated using an ITT approach (including all data regardless of adherence to treatment); missing lipid data were accounted for using a mixed-effects model with repeated measures approach, except for lipoprotein(a) (Lp[a]) and TGs, which were analyzed using multiple imputation followed by robust regression.

The statistical methods used to analyze other lipid parameters and the percentage change in LDL-C from baseline to week 24 for each study pool, stratified by age and HeFH genetic confirmation status, are described in more detail in the supplementary materials.

## Results

### Baseline Patient Characteristics

In total, pooled data from 1257 randomized patients with HeFH were analyzed (FH I (*n* = 486); FH II (*n* = 249); HIGH FH (*n* = 107); and LONG TERM (*n* = 415)). Baseline characteristics of the pooled study populations (patients receiving alirocumab 75/150 mg Q2W in the FH I and FH II trials; patients receiving alirocumab 150 mg Q2W in the LONG TERM and HIGH FH trials; and patients receiving placebo in all four trials) according to age group are shown in Table 1. All patients included in this analysis were receiving statin treatment, with the majority across study and age groups receiving high-intensity statin (63.2–87.8%). Baseline characteristics were similar between treatment groups across all age groups; the proportion of males decreased, whereas the proportion with CHD, diabetes, hypertension, and declining renal function increased with age.

**Table 1:** Baseline patient characteristics according to age group (randomized population)

	ALI 75/150 mg Q2W^a^				ALI 150 mg Q2W^b^				PBO^c^			
Age in years (n)	18 to < 45 (*n* = 132)	≥ 45 to < 55 (*n* = 129)	≥ 55 to < 65 (*n* = 139)	≥ 65 (*n* = 90)	18 to < 45 (*n* = 76)	≥ 45 to < 55 (*n* = 111)	≥ 55 to < 65 (*n* = 103)	≥ 65 (*n* = 58)	18 to < 45 (*n* = 103)	≥ 45 to < 55 (*n* = 109)	≥ 55 to < 65 (*n* = 138)	≥ 65 (*n* = 69)
Gender, male, n (%)	89 (67.4)	78 (60.5)	67 (48.2)	32 (35.6)	45 (59.2)	59 (53.2)	55 (53.4)	26 (44.8)	64 (62.1)	59 (54.1)	71 (51.4)	34 (49.3)
Race, White, n (%)	126 (95.5)	123 (95.3)	130 (93.5)	85 (94.4)	72 (94.7)	105 (94.6)	98 (95.1)	57 (98.3)	90 (87.4)	103 (94.5)	129 (93.5)	66 (95.7)
BMI, kg/m^2^, mean (SD)	28.5 (4.8)	29.3 (4.3)	28.9 (4.7)	28.5 (4.6)	28.5 (5.6)	30.4 (6.5)	27.9 (3.8)	30.1 (5.3)	28.5 (4.8)	30.2 (6.0)	29.1 (4.4)	29.0 (5.3)
Patients on statin, n (%)	132 (100.0)	129 (100.0)	139 (100.0)	90 (100.0)	76 (100.0)	111 (100.0)	103 (100.0)	58 (100.0)	103 (100.0)	109 (100.0)	138 (100.0)	69 (100.0)
High-intensity statin, *n* (%)^d^	108 (81.8)	109 (84.5)	122 (87.8)	73 (81.1)	48 (63.2)	82 (73.9)	79 (76.7)	42 (72.4)	81 (78.6)	85 (78.0)	110 (79.7)	60 (87.0)
eGFR, ml/min/1.73m^2^, mean (SD)	90.2 (17.5)	84.4 (13.9)	78.0 (15.4)	68.0 (16.7)	88.5 (12.1)	82.1 (14.8)	80.3 (13.3)	71.5 (17.5)	90.1 (19.7)	82.2 (17.3)	77.7 (20.1)	72.2 (16.9)
CHD, *n* (%)	28 (21.2)	46 (35.7)	73 (52.5)	58 (64.4)	22 (28.9)	41 (36.9)	54 (52.4)	29 (50.0)	21 (20.4)	48 (44.0)	83 (60.1)	44 (63.8)
Diabetes, *n* (%)	1 (0.8)	10 (7.8)	12 (8.6)	16 (17.8)	1 (1.3)	16 (14.4)	16 (15.5)	14 (24.1)	7 (6.8)	13 (11.9)	22 (15.9)	13 (18.8)
Baseline lipid parameters												
LDL-C (calculated), mg/dl, mean (SD)	150.4 (51.7)	146.7 (49.8)	135.8 (40.7)	128.6 (48.1)	190.7 (74.8)	168.3 (49.7)	161.4 (56.3)	150.3 (47.5)	159.5 (50.1)	151.1 (52.7)	147.4 (51.4)	137.0 (47.7)
mmol/l, mean (SD)	3.89 (1.34)	3.80 (1.29)	3.52 (1.05)	3.33 (1.25)	4.94 (1.94)	4.36 (1.29)	4.18 (1.46)	3.89 (1.23)	4.13 (1.30)	3.91 (1.37)	3.82 (1.33)	3.55 (1.23)
Non-HDL-C, mg/dl, mean (SD)	174.5 (55.0)	172.3 (54.4)	161.1 (43.7)	154.5 (51.8)	217.0 (79.2)	196.5 (53.4)	187.5 (61.2)	178.0 (53.2)	183.9 (55.5)	178.5 (55.3)	172.7 (54.2)	162.7 (51.0)
mmol/l, mean (SD)	4.52 (1.42)	4.46 (1.41)	4.17 (1.13)	4.00 (1.34)	5.62 (2.05)	5.09 (1.38)	4.86 (1.58)	4.61 (1.38)	4.76 (1.44)	4.62 (1.43)	4.47 (1.40)	4.21 (1.32)
HDL-C, mg/dl, mean (SD)	48.4 (14.2)	49.0 (15.6)	53.8 (16.3)	55.7 (15.7)	45.2 (14.7)	50.2 (12.2)	52.0 (11.1)	52.2 (12.1)	44.8 (13.4)	49.4 (14.9)	52.6 (13.2)	53.2 (14.7)
mmol/l, mean (SD)	1.25 (0.37)	1.27 (0.40)	1.39 (0.42)	1.44 (0.41)	1.17 (0.38)	1.30 (0.32)	1.35 (0.29)	1.35 (0.31)	1.16 (0.35)	1.28 (0.39)	1.36 (0.34)	1.38 (0.38)
Triglycerides, mg/dl, median (Q1:Q3)	106.0 (74.0:147.0)	112.0 (82.0:149.0)	111.0 (81.0:155.0)	111.0 (86.0:144.0)	105.2 (78.3:151.3)	128.0 (87.6:170.8)	115.0 (86.7:147.0)	127.4 (89.4:177.9)	104.0 (71.0:167.0)	112.1 (89.0:165.0)	111.3 (86.0:155.0)	108.0 (93.0:149.6)
mmol/l, median (Q1:Q3)	1.20 (0.84:1.66)	1.27 (0.93:1.68)	1.25 (0.92:1.75)	1.25 (0.97:1.63)	1.19 (0.89:1.71)	1.45 (0.99:1.93)	1.30 (0.98:1.66)	1.44 (1.01:2.01)	1.18 (0.80:1.89)	1.27 (1.01:1.86)	1.26 (0.97:1.75)	1.22 (1.05:1.69)
ApoB, mg/dl, mean (SD)	116.4 (32.3)	116.7 (30.9)	109.1 (26.7)	104.3 (27.0)	136.4 (42.2)	128.1 (29.5)	123.6 (35.1)	116.5 (27.6)	122.0 (32.8)	118.6 (31.1)	115.5 (30.4)	108.1 (26.2)
Lp(a), mg/dl, median (Q1:Q3)	25.0 (8.0:71.0)	41.0 (16.0:82.0)	26.0 (9.0:83.0)	29.0 (11.0:80.0)	23.1 (9.4:49.0)	19.0 (6.9:56.2)	31.7 (12.6:81.6)	26.0 (11.0:63.2)	26.0 (7.0:78.3)	22.0 (5.0:77.6)	20.9 (8.0:68.0)	32.5 (13.2:79.5)
Total cholesterol, mg/dl, mean (SD)	222.9 (55.1)	221.3 (54.4)	214.8 (45.6)	210.2 (51.1)	261.8 (79.9)	246.6 (52.1)	239.5 (62.2)	229.8 (53.3)	228.7 (55.3)	228.0 (55.9)	225.3 (53.2)	215.9 (51.4)
Total cholesterol, mmol/l, mean (SD)	5.77 (1.43)	5.73 (1.41)	5.56 (1.18)	5.44 (1.32)	6.78 (2.07)	6.39 (1.35)	6.20 (1.61)	5.95 (1.38)	5.92 (1.43)	5.90 (1.45)	5.84 (1.38)	5.59 (1.33)
ApoA1, mg/dl, mean (SD)	136.9 (25.4)	139.9 (27.4)	149.5 (30.1)	152.0 (26.7)	129.3 (31.0)	143.9 (25.3)	147.1 (23.0)	148.7 (21.3)	130.0 (28.7)	141.6 (29.1)	148.5 (26.7)	148.8 (25.3)

^a^Pool of two trials (FH I, NCT01623115; FH II, NCT01709500)

^b^Pool of two trials (LONG TERM, NCT01507831; HIGH FH, NCT01617655)

^c^Pool of all four trials (FH I, FH II, LONG TERM, HIGH FH)

^d^High-intensity statin corresponds to atorvastatin 40–80 mg, rosuvastatin 20–40 mg, or simvastatin 80 mg per day

ALI, alirocumab; Apo, apolipoprotein; BMI, body mass index; CHD, coronary heart disease; eGFR, estimated glomerular filtration rate; HDL-C, high-density lipoprotein cholesterol; HeFH, heterozygous familial hypercholesterolemia; LDL-C, low-density lipoprotein cholesterol; Lp(a), lipoprotein (a); non-HDL-C, non-high-density lipoprotein cholesterol; PBO, placebo; Q2W, every 2 weeks; SD, standard deviation

Baseline LDL-C, apolipoprotein (apo) B, non-high-density lipoprotein cholesterol (non-HDL-C), and total cholesterol decreased with age across study pools. Baseline high-density lipoprotein cholesterol (HDL-C) and apoA1 increased with age across all study pools. No age-related trends were observed for baseline TGs or Lp(a) (Table 1).

### Efficacy

#### Impact of Age on LDL-C Response

**Fig. 1:**
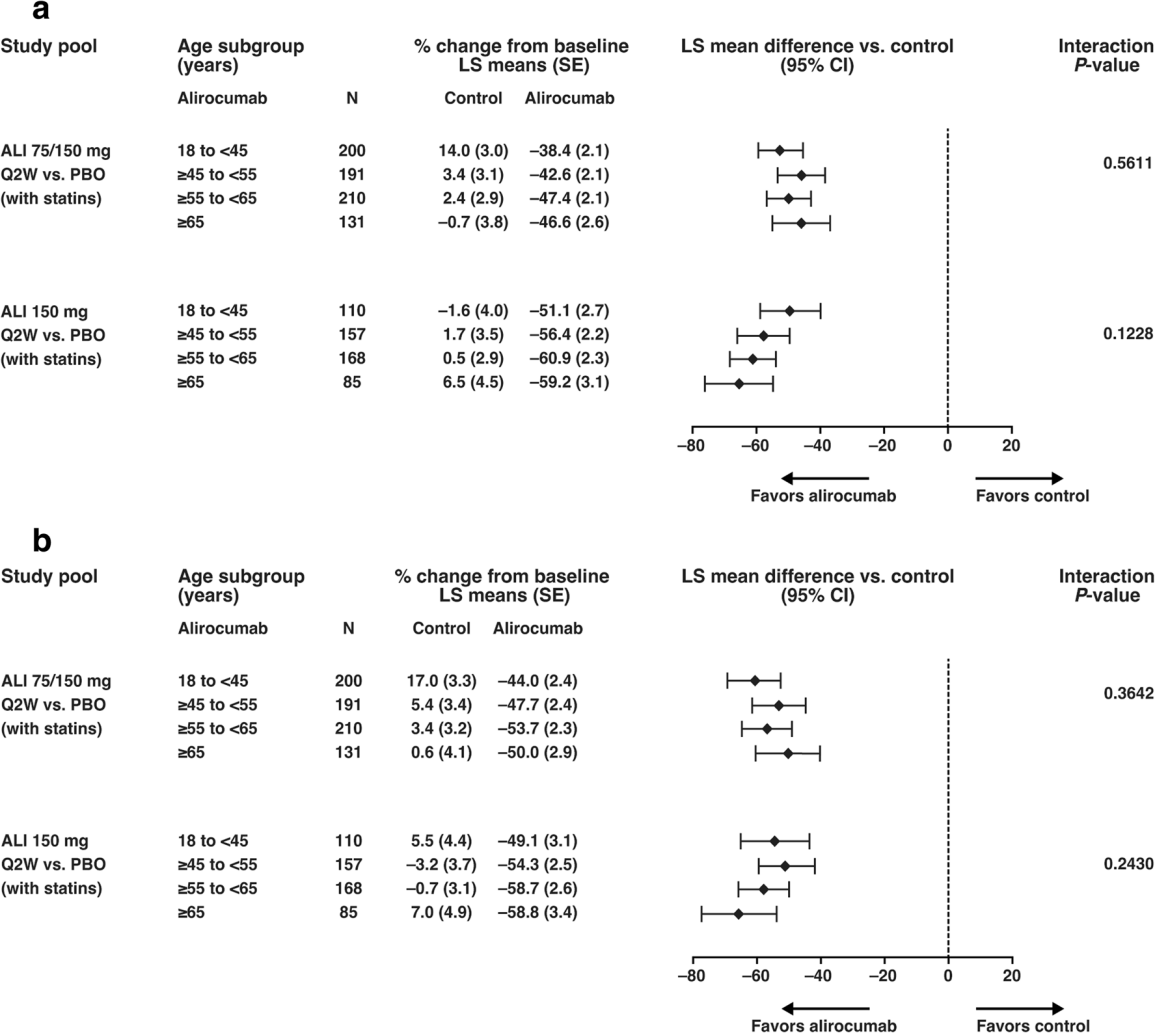
Subgroup analysis of percentage change in LDL-C from baseline to (A) week 12 and (B) week 24 for each study pool stratified by age (ITT analysis). LS means, SEs, and *P*-values were taken from mixed-effects model with repeated measures analysis. The model includes the fixed categorical effects of treatment group, time point, age group, randomization strata (as per IVRS), treatment-by-time point interaction, age group-by-time point interaction, treatment-by-age group interaction, and treatment-by-age group-by-time point interaction, as well as the continuous fixed covariates of specified baseline lipids parameter value and baseline value-by-time point interaction. Interaction *P*-value was calculated from the contrasts of interaction test treatment-by-age group at each visit. ALI, alirocumab; CI, confidence interval; ITT, intent-to-treat; IVRS, interactive voice response system, LDL-C, low-density lipoprotein cholesterol; LS, least squares; PBO, placebo; Q2W, every 2 weeks; SE, standard error

For all age groups, patients in the ITT population receiving alirocumab showed significant reductions in LDL-C compared with placebo from baseline to week 12 and week 24 (Fig. 1). At week 12 (Fig. 1a), before any potential dose increase in the 75/150 mg Q2W dose group, LDL-C reductions from baseline (least squares [LS] mean difference vs. placebo) were − 45.9% to −52.5% (alirocumab 75/150 mg Q2W) and − 49.5% to −65.6% (alirocumab 150 mg Q2W). At week 12, 41.8% of patients on alirocumab 75 mg Q2W had their dose increased in a blinded manner to 150 mg Q2W [10]. At week 24 (Fig. 1b), LDL-C reductions from baseline (LS mean difference vs. placebo) were − 50.6% to −61.0% (alirocumab 75/150 mg Q2W) and − 49.5% to −65.6% (alirocumab 150 mg Q2W). LDL-C response to alirocumab was consistent between age groups at week 12 in the alirocumab 75/150 mg Q2W pool (interaction *P*-value = 0.5611) or alirocumab 150 mg Q2W (interaction *P*-value = 0.1228) group, and week 24 in the alirocumab 75/150 mg Q2W pool (interaction *P*-value = 0.3642) or alirocumab 150 mg Q2W group (interaction *P*-value = 0.2430; Fig. 1).

#### Impact of Age and HeFH Genetic Confirmation Status on LDL-C Response

Just under half of the patients (44.6%) had genetically confirmed HeFH. As shown in Supplementary Fig. S1, LDL-C reductions were consistent whether diagnosis of HeFH was by genetic versus clinical criteria in the alirocumab 75/150 mg versus placebo group (interaction *P*-value = 0.4959), and the alirocumab 150 mg versus placebo group (interaction *P*-value = 0.2453) regardless of age.

#### Impact of Age on Other Lipid Parameters

Changes from baseline in other lipid parameters at week 24 by age subgroups are shown in Supplementary Table S1. No differences in response to alirocumab according to age were observed for other lipid parameters (total cholesterol, apoB, non-HDL-C, HDL-C, TGs, and Lp(a)) assessed at week 24 in either of the study pools (Supplementary Table S1).

### Safety by Age Group

The rates of reported TEAEs were comparable between alirocumab and control across all age subgroups, except for injection-site reactions which were more common in alirocumab than placebo but declined in frequency with age (8.1% of patients aged ≥ 65 years vs. 14.4% in patients aged 18 to < 45 years) (Supplementary Table S2).

LS mean glycated hemoglobin (HbA_1c_) levels at baseline were 5.4–5.9% across all age groups and treatment groups, while LS mean fasting plasma glucose (FPG) levels at baseline were 92.5–105.9 mg/dl across all age and treatment groups (Supplementary Table S3). Mean HbA_1c_ and FPG levels over the treatment period showed that alirocumab had no clinically meaningful effect on these parameters compared to placebo, regardless of age (Supplementary Figs. S2 and S3). The proportions of patients without diabetes at baseline who then developed diabetes were (for alirocumab vs. placebo): 1% versus 0%, 2.1% versus 3.7%, 4.6% versus 6.5%, and 2.0% versus 5.8%, for those aged 18 to < 45, ≥ 45 to < 55, ≥ 55 to < 65, and ≥ 65 years, respectively.

## Discussion

In this analysis, younger patients with HeFH (those aged 18 to < 45 years) had generally higher baseline LDL-C than older patients (≥ 65 years), despite receiving maximally tolerated statin. While the reasons for this difference are uncertain, there are several possibilities: older FH patients with high LDL-C levels might be under-represented because they had died of CVD; younger patients might have been enrolled by their physicians only if they had higher LDL-C levels; and younger patients in this group could have been less adherent to medication prior to involvement in the trial. In a previous study, statin adherence was poor in younger patients with HeFH (≤ 40 years) [11]. The proportion of participants with genetic confirmation of HeFH was 44.6% based on medical records. For the other 55.4%, HeFH diagnosis was based on clinical classification. This does not necessarily mean that the patients diagnosed by clinical criteria did not have a detectable mutation; they may not have been genotyped. Of note, a separate post-hoc analysis, which included a subset of participants from the ODYSSEY FH I, FH II, LONG TERM, and HIGH FH trials who consented to sequencing, examined the influence of genotype on treatment responses with alirocumab using Sanger sequencing [12]. Despite this approach, 24.6% of patients had no detectable mutation.

Alirocumab significantly reduced LDL-C levels in patients with HeFH compared with placebo, in agreement with the previous results [10], and no significant age differences in response to alirocumab were observed at weeks 12 and 24 across the age subgroups studied for LDL-C or other lipid parameters. Method of HeFH diagnosis (genetic or clinical) also did not modify LDL-C reductions. Similarly, age did not have an impact on frequency of TEAEs across the age groups studied, and alirocumab was generally well tolerated across all groups.

LDL-C reductions and safety results with alirocumab in HeFH patients were consistent with those observed with another PCSK9 inhibitor, evolocumab; a pooled analysis of the 48-week RUTHERFORD (*n* = 147, NCT01375751) and RUTHERFORD-2 (*n* = 293, NCT01763918) trials has established the long-term safety and efficacy of evolocumab in patients with HeFH [13]. For older patients, guidelines suggest that lipid-lowering therapies should be continued in those with dyslipidemia to reduce the risk of CVD. The ESC/EAS 2016 guidelines recommend treatment with statins in older adults with established CVD, and that statin therapy should also be considered in older adults free from CVD (especially patients with hypertension, diabetes, or dyslipidemia, or those who smoke) [4]. The 2016 American College of Cardiology Expert Consensus Decision Pathway also states that although fewer patients aged > 75 years were included in clinical trials compared with other groups, available evidence supports continuation of treatment with statins in patients aged > 75 years who are already receiving and tolerating statins [6]. The FOURIER trial showed that evolocumab reduced the risk of CV events in patients with CVD; the mean age of patients was 62.5 years, and in addition more than 12,000 individuals in this study were ≥ 65 years old [14].

Alirocumab treatment did not modify glycemic parameters (i.e., HbA_1c_ and FPG), with similar results observed for all age groups. In addition, very low numbers of patients with new-onset diabetes were observed, although there were insufficient patient numbers for formal statistical analyses. A recent pre-specified analysis of the ODYSSEY OUTCOMES trial found no evidence of new-onset diabetes in patients without diabetes treated with alirocumab [15].

A limitation of this analysis was the limited sample sizes per age group, specifically in older patients (e.g. patients > 75 years of age). Also, no patients were recruited aged < 18 years, hence no conclusions can be made about such younger patients. Of note, a trial of alirocumab in children and adolescents with HeFH is underway (clinicaltrials.gov: NCT02890992).

In conclusion, treatment with alirocumab (75 mg and 150 mg Q2W) led to significant LDL-C reductions, which is consistent with the previous findings [10]. Age did not significantly modify LDL-C response to alirocumab, or other lipid parameters, and alirocumab was generally well tolerated in patients with HeFH across all age groups studied, including older patients. These findings support the use of alirocumab across all age groups in patients aged ≥ 18 years with HeFH.

## Electronic supplementary material

ESM 1 (DOCX 331 kb)
