# Supplementary material for: Impact of Age on the Efficacy and Safety of Alirocumab in Patients with Heterozygous Familial Hypercholesterolemia
Source: Cardiovasc Drugs Ther. 2019 Feb 8;33(1):69–76. doi: 10.1007/s10557-019-06852-6 (PMC6433806; doi:10.1007/s10557-019-06852-6)
Supplement: Supplementary file 1 — (DOCX 331 kb) [file 10557_2019_6852_MOESM1_ESM.docx]

# Supplementary Material

Table S1. Subgroup analysis of percentage change in other lipid parameters from baseline to Week 24 for each study pool according to age group (ITT analysis)^a^

|  | Age group (years) | ApoB LS mean difference (SE) vs. PBO | Non-HDL-C LS mean difference (SE) vs. PBO | TC LS mean difference (SE) vs. PBO | Lp(a) adjusted mean difference (SE) vs. PBO | Fasting TGs adjusted mean difference (SE) vs. PBO | HDL-C LS mean difference (SE) vs. PBO | ApoA1 LS mean difference (SE) vs. PBO |
| --- | --- | --- | --- | --- | --- | --- | --- | --- |
| ALI 75/150 mg Q2W vs. PBO | 18 to <45 | –48.9 (3.0) | –55.7 (3.7) | –42.7 (2.8) | –15.7 (3.8) | –14.1 (4.1) | 6.6 (2.3) | 3.7 (1.9) |
|  | ≥45 to <55 | –40.2 (3.1) | –47.8 (3.8) | –35.4 (2.9) | –15.6 (3.9) | –18.3 (4.2) | 8.0 (2.4) | 4.7 (2.0) |
|  | ≥55 to <65 | –44.0 (2.9) | –51.4 (3.5) | –37.3 (2.7) | –24.5 (3.6) | –13.6 (3.9) | 5.6 (2.2) | 3.3 (1.8) |
|  | ≥65 | –39.8 (3.8) | –44.7 (4.6) | –29.5 (3.5) | –16.9 (4.6) | –10.7 (5.0) | 12.4 (2.9) | 8.1 (2.4) |
| ALI 150 mg Q2W vs. PBO | 18 to <45 | –43.8 (4.8) | –49.5 (4.9) | –38.1 (3.9) | –21.7 (5.5) | –24.3 (6.6) | 11.4 (3.2) | 10.3 (3.3) |
|  | ≥45 to <55 | –44.1 (4.0) | –46.7 (4.1) | –36.1 (3.3) | –22.7 (4.8) | –10.4 (5.5) | 2.0 (2.7) | 0.3 (2.8) |
|  | ≥55 to <65 | –47.9 (3.7) | –51.2 (3.8) | –37.8 (3.0) | –22.2 (4.2) | –12.3 (5.0) | 5.1 (2.4) | 6.5 (2.5) |
|  | ≥65 | –51.7 (5.4) | –57.2 (5.5) | –43.5 (4.4) | –30.3 (6.2) | –15.1 (7.3) | 0 (3.6) | –0.1 (3.7) |

^a^ Mixed effect model with repeated measures analysis; Lp(a) and TGs were analyzed using multiple imputation followed by robust regression.

All interaction *P*-values were non-significant.

For ApoB, non-HDL-C, TC, HDL-C, and ApoA1, LS means, SEs, and *P*-values were taken from mixed-effect model with repeated measures analysis. The model includes the fixed categorical effects of treatment group, time point, age group, randomization strata (as per IVRS), treatment-by-time point interaction, age group-by-time point interaction, treatment-by-age group interaction, and treatment-by-age group-by-time point interaction, as well as the continuous fixed covariates of specified baseline lipids parameter value and baseline value-by-time point interaction. Interaction *P-*value was from overall treatment-by-age group-by-time point interaction.

For Lp(a) and TGs, multiple imputation method was used to address missing values. Adjusted means and SEs were obtained by combining adjusted means and SEs from robust regression model analyses of the different imputed data sets. The robust regression models include the fixed categorical effect of treatment group, randomization strata as per IVRS, and the continuous fixed covariate of specified baseline lipids parameter value. Rubin's formulae were used to combine means and SEs.

ALI, alirocumab; Apo, apolipoprotein; HDL-C, high-density lipoprotein cholesterol; ITT, intent-to-treat; IVRS, Interactive Voice Response System, Lp(a), lipoprotein(a); LS, least squares; non-HDL-C, non-high-density lipoprotein cholesterol; PBO, placebo; Q2W, every 2 weeks; SE, standard error; TC, total cholesterol; TGs, triglycerides

Table S2. Safety summary by age group (safety population)

|  | 18 to <45 years | | ≥45 to <55 | | ≥55 to <65 | | ≥65 | |
| --- | --- | --- | --- | --- | --- | --- | --- | --- |
| ***n* (%)** | **ALI (*n* = 208)** | **PBO (*n* = 102)** | **ALI (*n* = 240)** | **PBO (*n* = 109)** | **ALI (*n* = 241)** | **PBO (*n* = 138)** | **ALI (*n* = 148)** | **PBO (*n* = 69)** |
| TEAEs | 161 (77.4) | 83 (81.4) | 200 (83.3) | 92 (84.4) | 198 (82.2) | 112 (81.2) | 115 (77.7) | 60 (87.0) |
| Treatment-emergent SAEs | 16 (7.7) | 4 (3.9) | 34 (14.2) | 13 (11.9) | 36 (14.9) | 21 (15.2) | 28 (18.9) | 17 (24.6) |
| TEAEs leading to death | 0 | 0 | 2 (0.8) | 1 (0.9) | 4 (1.7) | 1 (0.7) | 1 (0.7) | 0 |
| TEAEs leading to treatment discontinuation | 7 (3.4) | 5 (4.9) | 7 (2.9) | 3 (2.8) | 8 (3.3) | 4 (2.9) | 11 (7.4) | 3 (4.3) |
| TEAEs reported in at least 5% of patients^a^ | | | | | | | | |
| Arthralgia | 5 (2.4) | 5 (4.9) | 18 (7.5) | 5 (4.6) | 14 (5.8) | 13 (9.4) | 10 (6.8) | 5 (7.2) |
| Back pain | 13 (6.3) | 4 (3.9) | 15 (6.3) | 4 (3.7) | 10 (4.1) | 11 (8.0) | 8 (5.4) | 4 (5.8) |
| Bronchitis | 8 (3.8) | 9 (8.8) | 13 (5.4) | 4 (3.7) | 17 (7.1) | 5 (3.6) | 7 (4.7) | 2 (2.9) |
| Diarrhea | 13 (6.3) | 2 (2.0) | 13 (5.4) | 3 (2.8) | 11 (4.6) | 4 (2.9) | 6 (4.1) | 5 (7.2) |
| Dizziness | 7 (3.4) | 2 (2.0) | 7 (2.9) | 3 (2.8) | 9 (3.7) | 7 (5.1) | 7 (4.7) | 7 (10.1) |
| Fatigue | 3 (1.4) | 0 | 14 (5.8) | 5 (4.6) | 7 (2.9) | 4 (2.9) | 3 (2.0) | 6 (8.7) |
| Gastroenteritis | 8 (3.8) | 6 (5.9) | 8 (3.3) | 3 (2.8) | 6 (2.5) | 5 (3.6) | 6 (4.1) | 2 (2.9) |
| Headache | 18 (8.7) | 12 (11.8) | 17 (7.1) | 6 (5.5) | 11 (4.6) | 6 (4.3) | 10 (6.8) | 6 (8.7) |
| Hypertension | 5 (2.4) | 1 (1.0) | 7 (2.9) | 3 (2.8) | 7 (2.9) | 9 (6.5) | 6 (4.1) | 4 (5.8) |
| Influenza | 20 (9.6) | 13 (12.7) | 26 (10.8) | 11 (10.1) | 24 (10.0) | 9 (6.5) | 13 (8.8) | 3 (4.3) |
| Injection-site reaction | 30 (14.4) | 13 (12.7) | 32 (13.3) | 11 (10.1) | 21 (8.7) | 8 (5.8) | 12 (8.1) | 4 (5.8) |
| Muscle spasms | 9 (4.3) | 1 (1.0) | 9 (3.8) | 7 (6.4) | 11 (4.6) | 7 (5.1) | 5 (3.4) | 1 (1.4) |
| Myalgia | 6 (2.9) | 7 (6.9) | 18 (7.5) | 6 (5.5) | 9 (3.7) | 10 (7.2) | 10 (6.8) | 3 (4.3) |
| Nasopharyngitis | 31 (14.9) | 16 (15.7) | 32 (13.3) | 11 (10.1) | 26 (10.8) | 21 (15.2) | 20 (13.5) | 7 (10.1) |
| Peripheral edema | 1 (0.5) | 1 (1.0) | 5 (2.1) | 0 | 4 (1.7) | 5 (3.6) | 2 (1.4) | 4 (5.8) |
| Osteoarthritis | 0 | 2 (2.0) | 6 (2.5) | 1 (0.9) | 3 (1.2) | 3 (2.2) | 9 (6.1) | 2 (2.9) |
| Sinusitis | 7 (3.4) | 5 (4.9) | 12 (5.0) | 6 (5.5) | 8 (3.3) | 4 (2.9) | 3 (2.0) | 1 (1.4) |
| Upper respiratory tract infection | 11 (5.3) | 9 (8.8) | 15 (6.3) | 5 (4.6) | 18 (7.5) | 12 (8.7) | 7 (4.7) | 4 (5.8) |
| Urinary tract infection | 10 (4.8) | 2 (2.0) | 12 (5.0) | 6 (5.5) | 8 (3.3) | 6 (4.3) | 13 (8.8) | 7 (10.1) |

^a^ At least 5% in the overall alirocumab or placebo group (irrespective of age group).

ALI, alirocumab; PBO, placebo; SAE, serious adverse event; TEAE, treatment-emergent adverse event.

Table S3. Baseline HbA1c and FPG according to age group and treatment group (safety population)

| **Characteristic** | **Age group, years** | **N** | **Alirocumab, LS mean (SE)** | **Placebo LS mean (SE)** |
| --- | --- | --- | --- | --- |
| HbA1c, % | 18 to <45 | 297 | 5.4 (0.04) | 5.4 (0.05) |
|  | ≥45 to <55 | 341 | 5.7 (0.05) | 5.7 (0.07) |
|  | ≥55 to <65 | 375 | 5.9 (0.05) | 5.9 (0.06) |
|  | ≥65 | 215 | 5.9 (0.06) | 5.9 (0.09) |
| FPG, mg/dL | 18 to <45 | 310 | 92.5 (1.05) | 94.2 (1.47) |
|  | ≥45 to <55 | 347 | 99.8 (1.46) | 98.5 (2.13) |
|  | ≥55 to <65 | 379 | 101.4 (1.62) | 102.5 (2.07) |
|  | ≥65 | 216 | 105.2 (2.07) | 105.9 (2.92) |

FPG, fasting plasma glucose; HbA1c, glycated hemoglobin; LS, least squares; SE, standard error.

Figure S1. Subgroup analysis of percentage change in LDL-C from baseline to Week 24 for each study pool stratified by age and per HeFH genetic confirmation status (ITT analysis). Diagnosis of FH was based on patients’ medical records (genetic confirmation in 44.6% and clinical diagnosis in 55.4%).


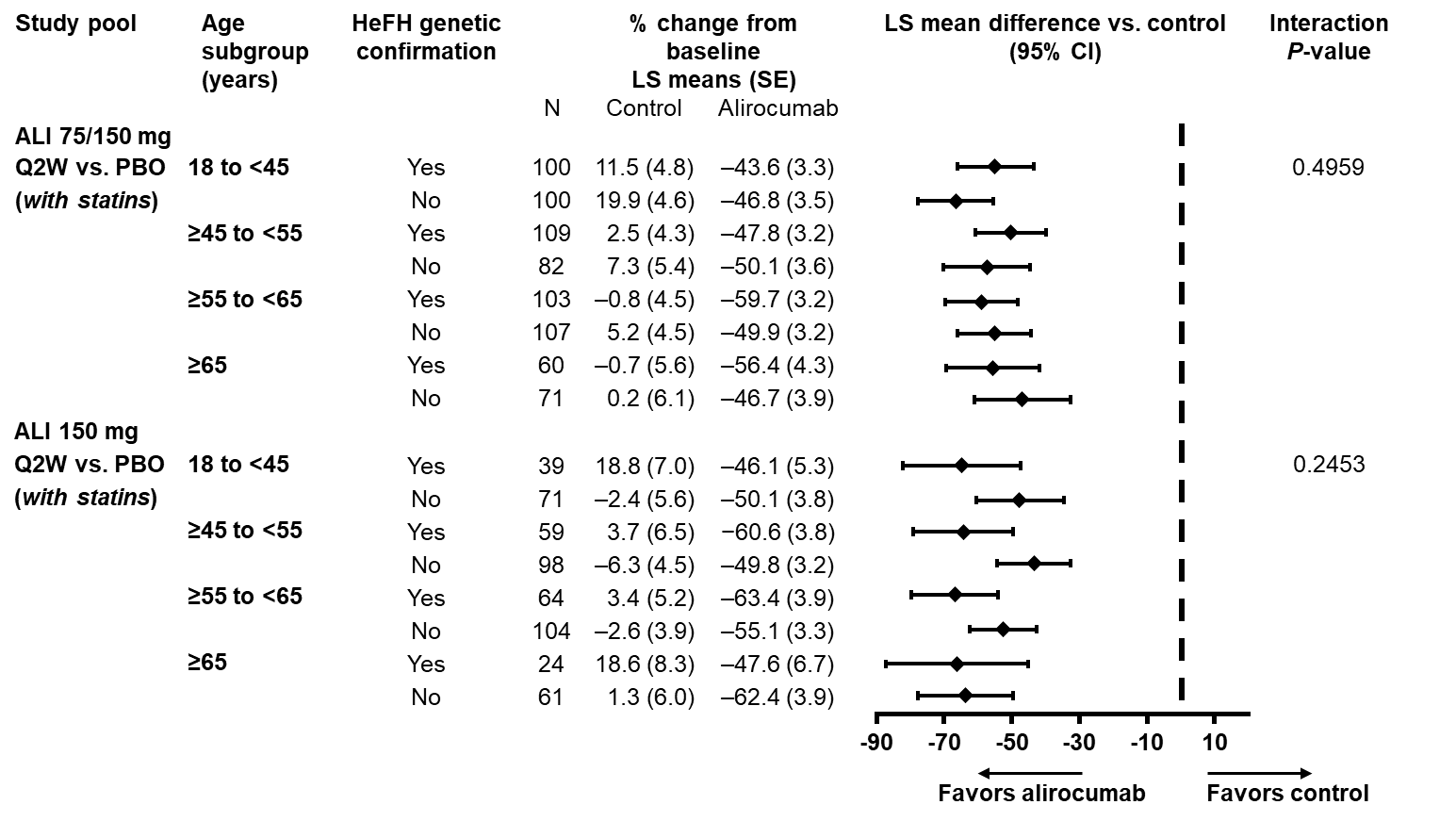


LS means, SEs, and *P*-values were taken from mixed-effect model with repeated measures analysis. The model includes the fixed categorical effects of treatment group, time point, age group, genotyping status, randomization strata (as per IVRS), treatment-by-time point interaction, age group-by-time point interaction, treatment-by-age group interaction, genotyping status-by-time point interaction, treatment-by-genotyping status interaction, and treatment-by-age group-by-genotyping status-by-time point interaction, as well as the continuous fixed covariates of specified baseline lipids parameter value and baseline value-by-time point interaction. Interaction *P*-value was from overall treatment-by-age group-by-genotyping status-by-time point interaction. **HeFH status was based on clinical diagnosis was based on either World Health Organization/Dutch Lipid Clinical Network criteria with a score >8 points, or meeting Simon Broome criteria for definite FH.**

ALI, alirocumab; CI, confidence interval; HeFH, heterozygous familial hypercholesterolemia; ITT, intention to treat; IVRS, Interactive Voice Response System; LDL-C, low-density lipoprotein cholesterol; LS, least squares; PBO, placebo; Q2W, every 2 weeks; SE, standard error

Figure S2. HbA_1c_ levels over time by age group: a) 18 to <45 years, b) ≥45 to <55 years, c) ≥55 to <65 years, and d) ≥65 years (safety population)


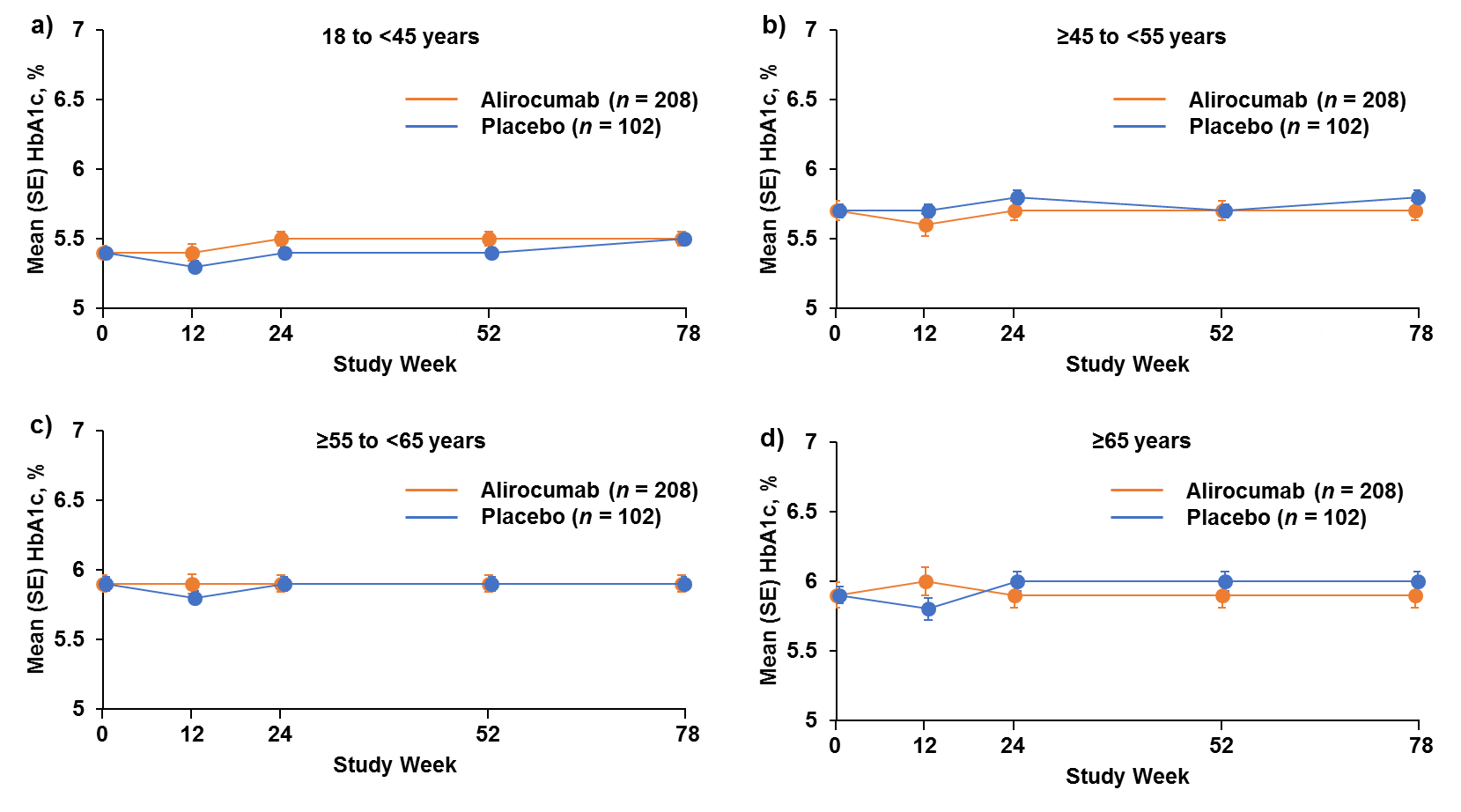


HbA1c, glycated hemoglobin; SE, standard error.

Figure S3. Fasting plasma glucose levels over time by age group: a) 18 to <45 years, b) ≥45 to <55 years, c) ≥55 to <65 years, and d) ≥65 years (safety population)


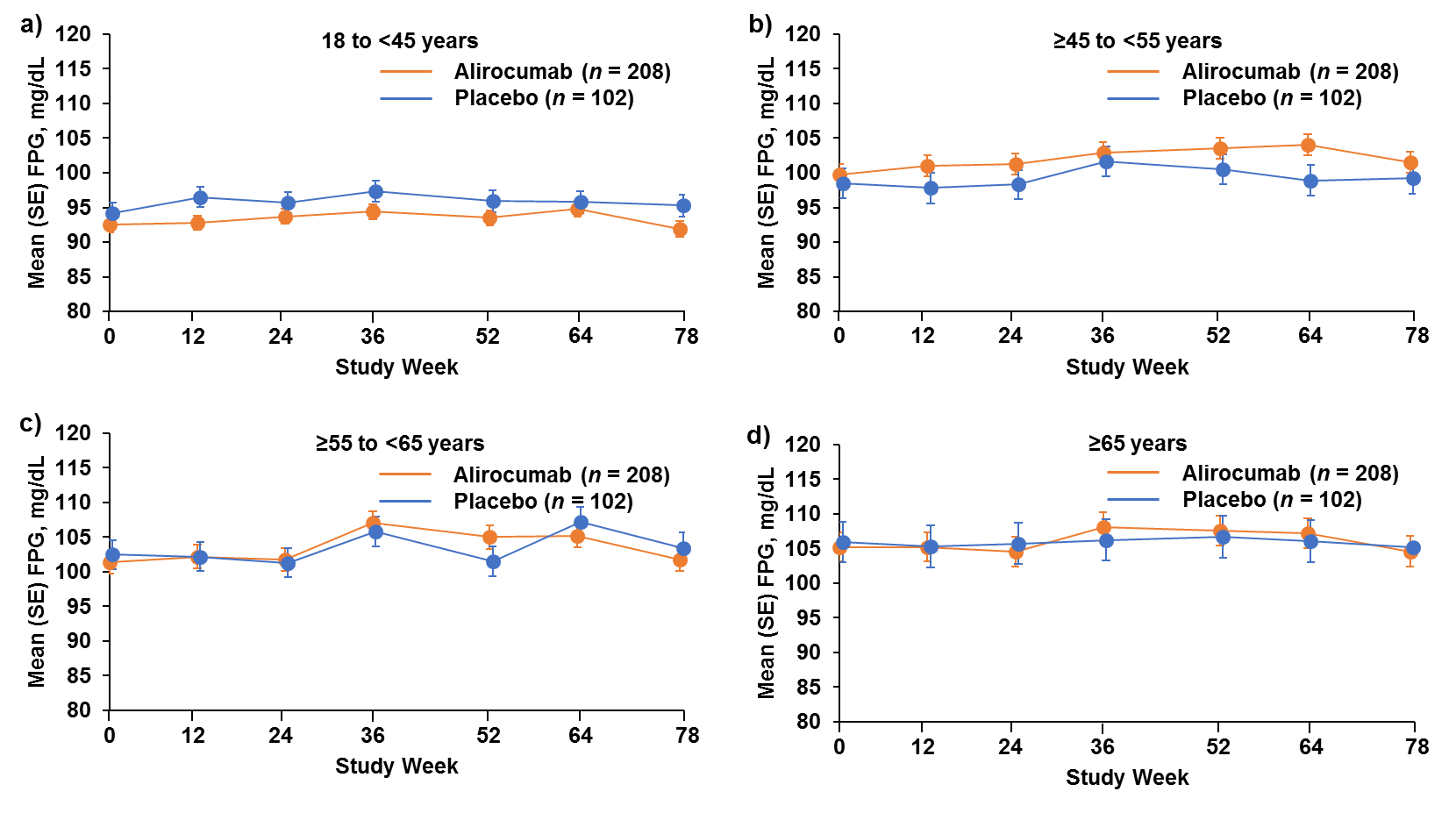


FPG, fasting plasma glucose; SE, standard error.
